# CT-measured body composition radiomics predict lymph node metastasis in localized pancreatic ductal adenocarcinoma

**DOI:** 10.1007/s12672-023-00624-3

**Published:** 2023-02-03

**Authors:** Qianbiao Gu, Mengqing He, Yaqiong He, Anqi Dai, Jianbin Liu, Xiang Chen, Peng Liu

**Affiliations:** Department of Radiology, Hunan Provincial People’s Hospital, First Affiliated Hospital of Hunan Normal University, Changsha, 410005 China

**Keywords:** Body composition, Radiomics analysis, Computed tomography, Lymph node metastasis, Pancreatic ductal adenocarcinoma

## Abstract

**Background:**

To explored the value of CT-measured body composition radiomics in preoperative evaluation of lymph node metastasis (LNM) in localized pancreatic ductal adenocarcinoma (LPDAC).

**Methods:**

We retrospectively collected patients with LPDAC who underwent surgical resection from January 2016 to June 2022. According to whether there was LNM after operation, the patients were divided into LNM group and non-LNM group in both male and female patients. The patient’s body composition was measured by CT images at the level of the L3 vertebral body before surgery, and the radiomics features of adipose tissue and muscle were extracted. Multivariate logistic regression (forward LR) analyses were used to determine the predictors of LNM from male and female patient, respectively. Sexual dimorphism prediction signature using adipose tissue radiomics features, muscle tissue radiomics features and combined signature of both were developed and compared. The model performance is evaluated on discrimination and validated through a leave-one-out cross-validation method.

**Results:**

A total of 196 patients (mean age, 60 years ± 9 [SD]; 117 men) were enrolled, including 59 LNM in male and 36 LNM in female. Both male and female CT-measured body composition radiomics signatures have a certain predictive power on LNM of LPDAC. Among them, the female adipose tissue signature showed the highest performance (area under the ROC curve (AUC), 0.895), and leave one out cross validation (LOOCV) indicated that the signature could accurately classify 83.5% of cases; The prediction efficiency of the signature can be further improved after adding the muscle radiomics features (AUC, 0.924, and the accuracy of the LOOCV was 87.3%); The abilities of male adipose tissue and muscle tissue radiomics signatures in predicting LNM of LPDAC was similar, AUC was 0.735 and 0.773, respectively, and the accuracy of LOOCV was 62.4% and 68.4%, respectively.

**Conclusions:**

CT-measured body composition Radiomics strategy showed good performance for predicting LNM in LPDAC, and has sexual dimorphism. It may provide a reference for individual treatment of LPDAC and related research about body composition in the future.

**Supplementary Information:**

The online version contains supplementary material available at 10.1007/s12672-023-00624-3.

## Introduction

Pancreatic cancer has a bleak prognosis, with a 5-year survival rate of less than 10% [1]. Based on the incidence of all malignant tumors, it ranks eighth among female patients and tenth among male patients. However, the total number of patients dying from pancreatic cancer is as high as third among all tumor-related causes of death, with the mortality rate increasing year by year [1, 2]. Surgical resection is the only cure for pancreatic cancer, with a 5-year survival rate of about 25% [3]. Accurate preoperative evaluation of lymph node status is the basis of personalized treatment of localized pancreatic ductal adenocarcinoma (LPDAC), which plays an important role in the treatment decision-making of patients. It has been reported that the 5-year survival rate of patients with lymph node metastasis (LNM) confirmed by postoperative pathology is similar to that of patients without surgical treatment, but that of patients without LNM is as high as 40% [4, 5]. Therefore, the prognosis of patients with different LN statuses varies greatly, who may require different extents of lymph node dissection or neoadjuvant therapy. NCCN guidelines recommend preoperative CT to evaluate the resectability of pancreatic cancer [6]. However, traditional CT imaging-based LNM evaluation methods have yielded disappointing results due to low sensitivity and specificity [7, 8]. Recently, radiomics has shown great potential in predicting LNM of pancreatic cancer [9–12]. However, these studies focus on the tumor itself, ignoring that pancreatic cancer is actually a systemic disease and a malignant tumor with metabolic heterogeneity. The change of amino acid, lipid metabolism and glucose in pancreatic cancer significantly affects tumor progression, from cell to microenvironment and even at the systemic level [13, 14].

Recently, tumor metabolic reprogramming has aroused great interest in researchers. Accumulating evidence supports the existence of dynamic changes in the metabolism of metastatic cells. A number of studies have found that fat-related metabolic changes are emerging factors in LNM [15–17], indicating an era of metabolic changes that make cancer cells into LNM. Computed tomography (CT) measured body composition, including subcutaneous and visceral fat and muscle, has been shown to be associated with systemic inflammatory immune and metabolic status. More importantly, it has been recognized as a predictor of many tumor and metabolic diseases [18–20].

So far, no attempt has been made to predict the status of LNM in patients with pancreatic cancer based on human body composition radiomics. This study aims to explore whether CT-measured body composition radiomics can predict LNM in patients with LPDAC and whether there are gender differences in this method (Additional file 1).

## Materials and methods

### Patient data

**Fig. 1:**
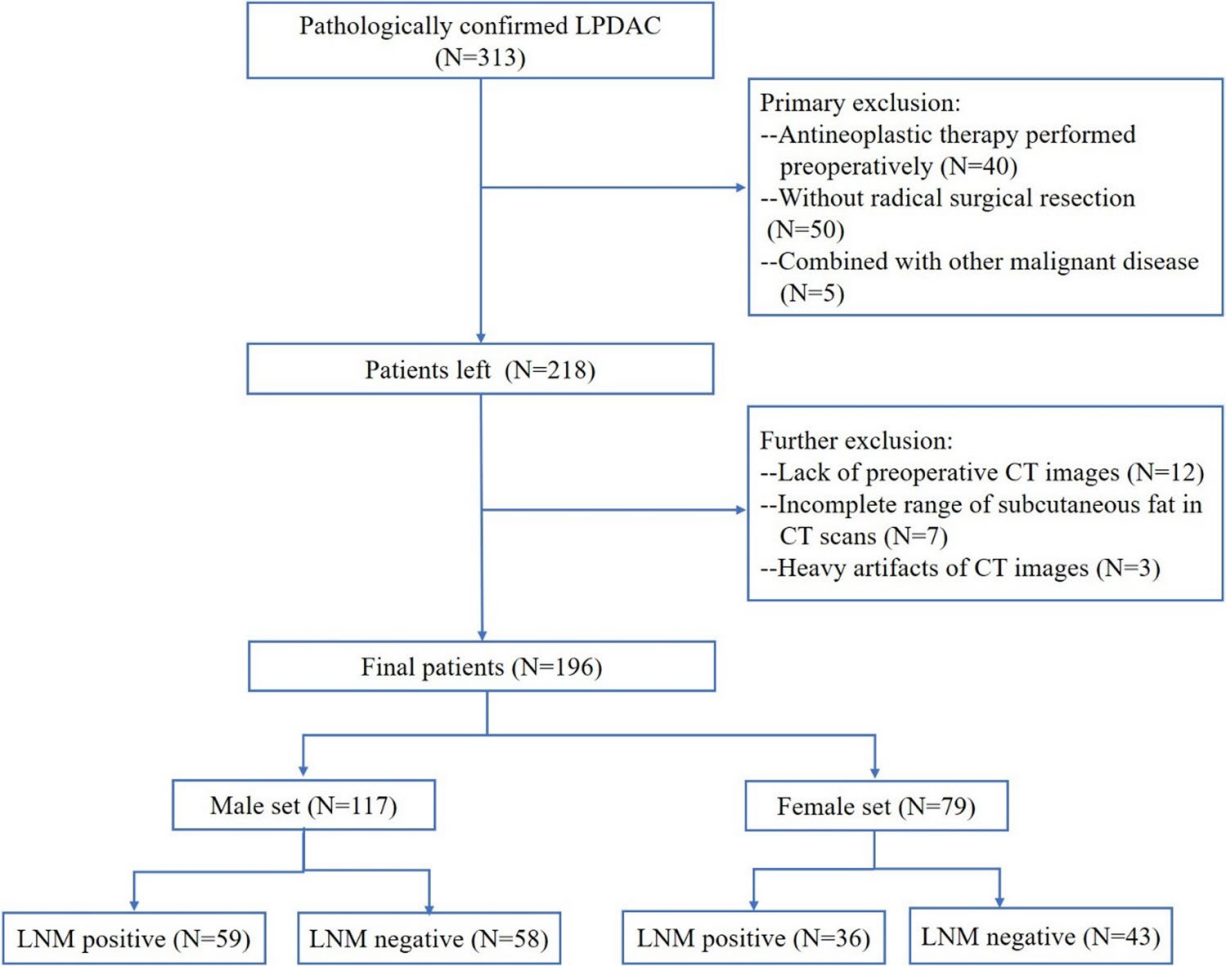
Patient flowchart for this study. *LPDAC* localized pancreatic ductal adenocarcinoma, *LNM* lymph node metastasis

This retrospective study received approval from the Institutional Review Board of our hospital, waiving the request for informed consent. Patients with LPDAC who accepted surgical resection in our hospital were retrospectively collected from January 2016 to June 2022. Inclusion criteria: 1. LPDAC was confirmed by postoperative pathology and immunohistochemistry examination. 2. A standard range of lymph node dissection was performed [21]. 3. CT scan was performed within 2 weeks before the operation. Exclusion criteria: 1. Antineoplastic therapy was performed preoperatively. 2. Patients with a history of other malignant diseases or concurrent primary cancers. 3. It is difficult to segment abdominal adipose tissue or skeletal muscle at the L3 level due to an incomplete scanning range of subcutaneous fat or excessive artifacts in CT images. The detailed process is shown in Fig. 1 This study included a total of 196 patients, including 79 females and 117 males.

### Image acquisition

All patients accepted non-enhanced and enhanced CT scans of the upper abdomen or the whole abdomen (including arterial phase and venous phase) with Philips Brilliance iCT 256 or Siemens Somatom Force CT machine before the operation. The contrast agent iohexol 300 (300 mg/ml) was injected at a speed of 3 ml/s and a dose of 1.2–1.5 ml/kg through the anterior vein of the right elbow with an UlrichXD 2060 double-barrel high pressure syringe. Arterial phase and venous phase scans were conducted at 25–35 s and 60–70 s after injection, respectively, with scanning thickness of 2 mm, reconstruction thickness of 5 mm, automatic pitch matching, tube voltage of 120 kV, and tube current of 200–380 mA.

### Body composition ROI segmentation

At present, most researchers have utilized the L3 vertebral body as the reference point for imaging-based body composition analysis [22, 23]. Therefore, body composition was measured by non-enhanced cross-sectional CT images at the L3 vertebral level to determine the region of adipose and muscle tissues. The adipose tissue included subcutaneous and visceral fat areas. Firstly, threshold-based ROI analysis was performed using the 3D Slicer software version 4.11.0 (www.slicer.org). Hounsfield unit (HU) thresholds were used as follows: adipose tissue from −190HU to −30HU; muscle tissue from −29HU to 150HU. Then, the mixed signals (such as intestinal content, caused by partial volume effect) were removed compared with the segmentation boundary of original CT images. It was segmented by two radiologists with 10 and 13 years of experience in abdominal imaging diagnosis, respectively.

### Radiomics feature extraction

On the 3DSlicer platform, radiomics features were extracted from each patient’s ROI using the open-source pyradiomic 3.0.1 version package. To obtain the isotropic voxels, ROI was resampled to 1 × 1 × 1 mm, and images were normalized to reduce the imaging differences between different CT scanning devices. To ensure better comparability of CT gray values, a fixed slot width of 25 was chosen. Before feature extraction, the normalized CT image was filtered by gradient, index, logarithm, square root, wavelet, log filtering and other built-in filtering to obtain the derived image. Finally, 1,688 radiomics features were extracted from each ROI. The extracted features consisted of adjacent gray difference matrix (NGTDM), gray run matrix (GLRLM), gray size region matrix (GLSZM), gray dependence matrix (GLDM), gray co-occurrence matrix (GLCM), two-dimensional feature, and first-order feature [24]. Radiomics features are detailed in Additional file 1.

### Radiomics feature selection and radiomics signature construction

**Fig. 2:**
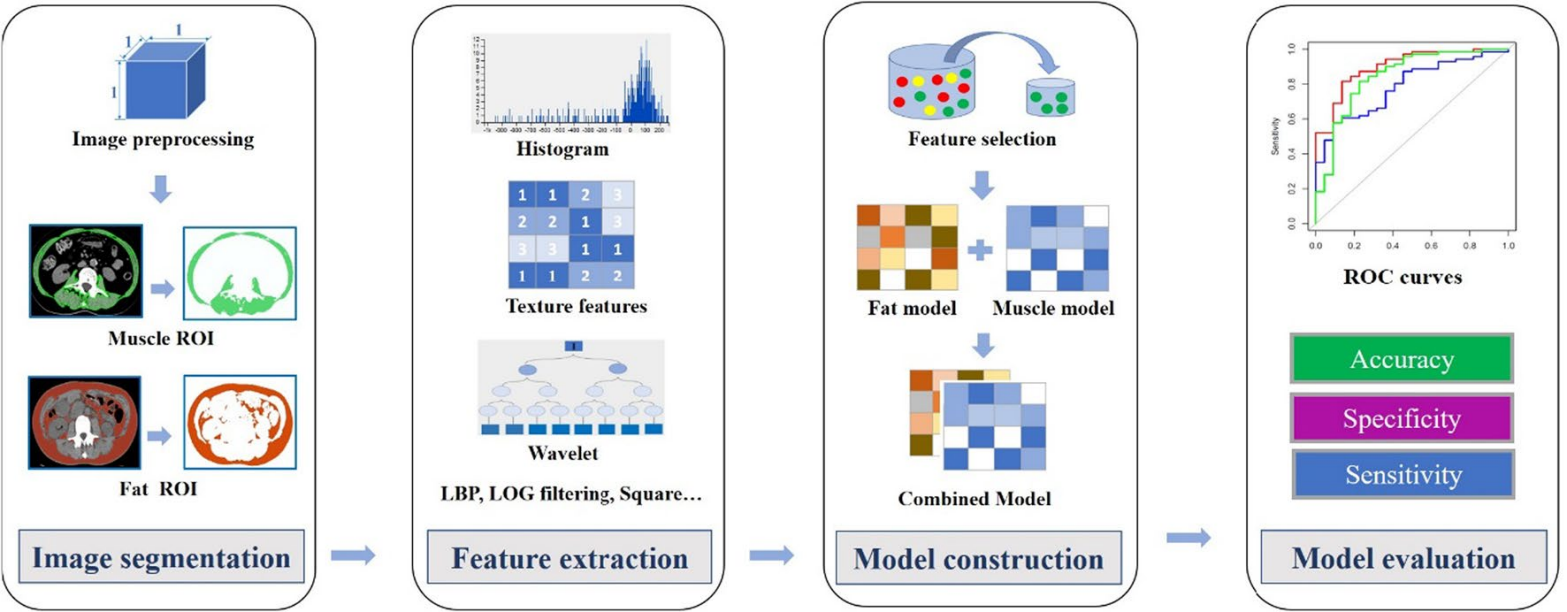
Rdiomics workflow and study flowchart. *ROI *region of interest, *ROC *Receiver Operating Characteristic

The R software Caret package was used to pre-process the data. Firstly, zero variance and near-zero variance radiomics features were identified and removed with nearZeroVar in the caret package. Then, based on the Spearman test, radiomics features with a correlation coefficient greater than 0.9 and multiple collinearities were deleted, and independent features were preliminarily selected. Subsequently, to reduce the impact of dimensionality on the data and improve the comparability of different features, the data normalization procedure was performed, including mean-centred division by the standard deviation of each feature. Finally, multivariate logistic regression (forward LR) was used to screen for independent predictors of adipose and muscle in the LNM of pancreatic cancer among male and female patients. Moreover, sex-specific adipose and muscle radiomics signatures were established according to the selected radiomics features and the corresponding weight coefficients. (Fig. 2).

### Statistical analysis

Statistical analysis was performed using IBM SPSS Statistics software (version 25) or R software (version 3.6.0; http://www.r-project.org). Continuous variables were represented as mean ± SD, and categorical variables were compared using the χ2 test. The area under the ROC curve (AUC), sensitivity and specificity were used to quantify the identification of the prediction signature. Leave one out cross validation was used to validate the prediction signature.

## Results

### Baseline characteristics

This study included a total of 196 patients, including 95 with LNM and 101 with non-LNM. Table 1 shows the basic clinical characteristics of these patients. No significant difference was found in sex, age, and longest tumor diameter between the LNM group and the non-LNM group (P > 0.05). There was a significant difference in the tumor location between the two groups (P = 0.03). It seemed that the tumor located in the pancreatic head was more prone to LNM.

**Table 1:** Baseline characteristics of patients with pancreatic ductal adenocarcinoma

Characteristic	LNM (n = 95)	Non-LNM (n = 101)	P value
Sex			0.50
Male	59 (62)	58 (57)	
Female	36 (38)	43 (43)	
Age*	60 ± 10	60 ± 9	0.47
Longest diameter*	3.56 ± 1.63	3.52 ± 1.76	0.67
Location			0.03
Body or tail	16 (17)	36 (35)	
Head	79 (83)	65 (65)	

Except where indicated, data are numbers of patients, with percentages in parentheses

*LNM* lymph node metastasis

^*^Data are means ± SDs

### Radiomics signature building

Among the 1688 radiomics features, 1211 features were retained after removing zero variance and near-zero variance radiomics features. Then, 329 independent radiomics features were selected through the correlation and collinearity tests. After splitting the cohort by gender, 5 radiomics features from adipose and 4 radiomics features from muscle were screened to predict LNM in male patients using the multivariate logistic regression (forward LR) method. The radiomics signature of male adipose with multivariate logistic regression was calculated using the following formula:

$$RSmale-fat=-3.790*gradient\_firstorder\_InterquartileRange+0.682*gradient\_glrlm\_RunLengthNonUniformity+0.658*gradient\_glszm\_SmallAreaLowGrayLevelEmphasis+1.475*lbp.2D\_firstorder\_10Percentile+0.655*wavelet.HHL\_glcm\_ClusterShade-0.453$$

The radiomics signature of male muscle with multivariate logistic regression was calculated using the following formula:

$$RSmale-muscle=1.035*wavelet.HHH\_firstorder\_Mean-0.743*wavelet.HHH\_firstorder\_Skewness+0.508*wavelet.HHH\_gldm\_LowGrayLevelEmphasis-0.749* wavelet.HHH\_glszm\_SmallAreaEmphasis+0.287$$

The sum of radiomics signature values of male muscle and adipose was used as a combined signature, namely:

$$RSmale-combined= RSmale-fat+ RSmale-muscle$$

Similarly, 7 radiomics features from adipose and 4 radiomics feature from muscle were screened to predict LNM in female patients using the multivariate logistic regression (forward LR) method. The radiomics signature of female adipose with multivariate logistic regression was calculated using the following formula:

$$RSfemale-fat=-2.201* logarithm\_glszm\_LargeAreaHighGrayLevelEmphasis-2.650*wavelet.HHL\_glcm\_Imc2+1.749*wavelet.HHL\_glszm\_LowGrayLevelZoneEmphasis+2.898*wavelet.HHL\_glszm\_ZoneEntropy-3.256*wavelet.HLH\_firstorder\_Median+1.630*wavelet.HLH\_glszm\_SmallAreaLowGrayLevelEmphasis-2.064*wavelet.HLL\_glcm\_Idmn-1.384$$

The radiomics signature of female muscle with multivariate logistic regression was calculated using the following formula:

$$RSfemale-muscle=1.002* wavelet.HHH\_firstorder\_Minimum+1.413*wavelet.HLH\_glszm\_GrayLevelVariance+0.633*wavelet.LHH\_glrlm\_LongRunLowGrayLevelEmphasis-0.996*wavelet.LHH\_glszm\_SmallAreaLowGrayLevelEmphasis-0.597$$

The sum of radiomics signature values of female muscle and adipose was used as a combined signature, namely:

$$RSfemale-combined= RSfemale-fat+ RSfemale-muscle$$

### Performance of radiomics signature

**Fig. 3:**
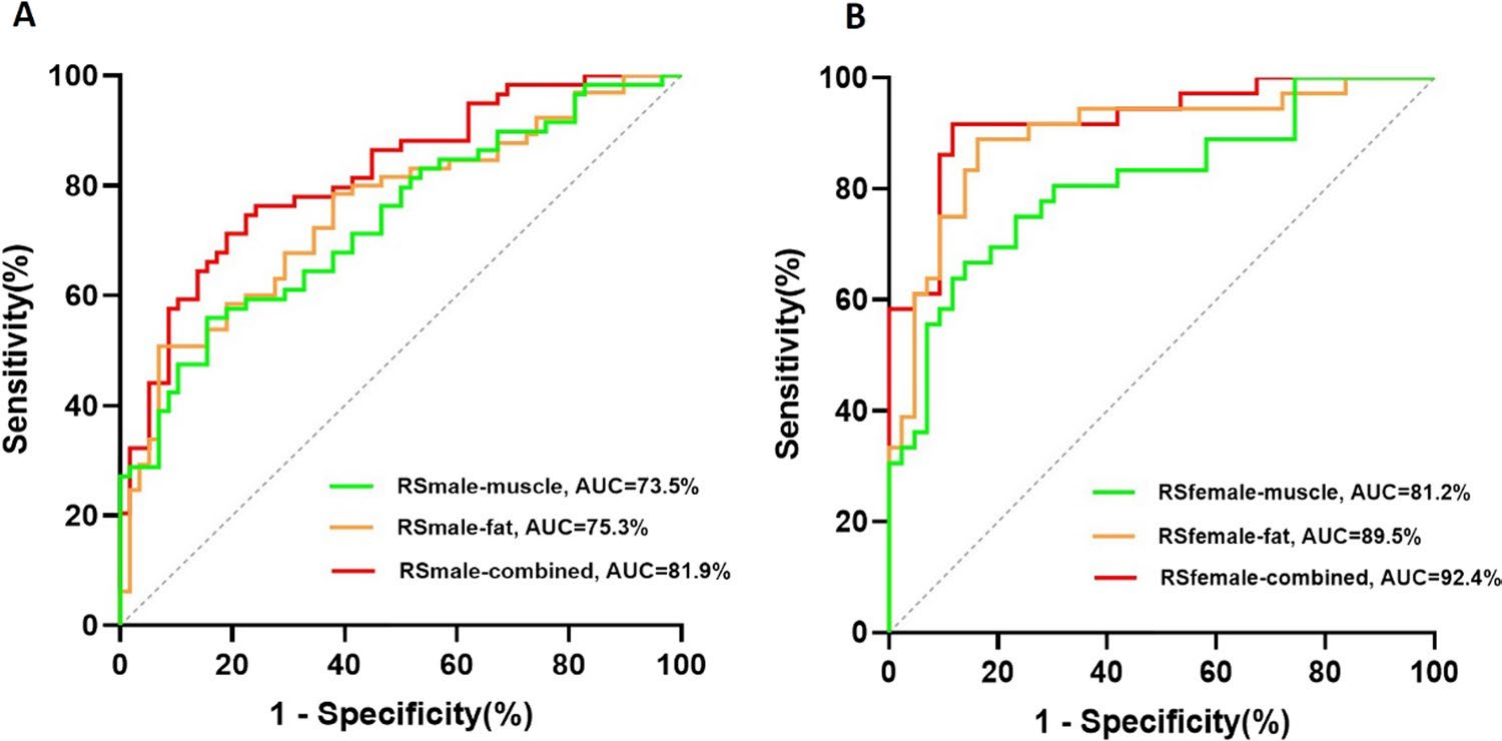
Performance of radiomics signature. *RS* radiomics score, *AUC* area under the curve

Both male and female CT-measured body composition radiomics signatures have a certain predictive power on LNM of LPDAC. For female patients, the AUC of adipose signature showed the highest performance at 0.895 (95% CI 0.821, 0.970), and leave one out cross validation (LOOCV) showed the signature could accurately classify 83.5% of cases. Moreover, the prediction efficiency of the signature can be further improved after adding the muscle radiomics features (AUC, 0.924 (95% CI 0.864, 0.984), and the accuracy of the LOOCV was 87.3%. For male patients, The prediction ability of adipose radiomics signature was similar to the radiomics signature of muscle, with an AUC of 0.735 (95% CI 0.645, 0.825), and 0.773 (95% CI 0.689, 0.858), respectively, and the accuracy of LOOCV was 62.4% and 68.4%, respectively. (Fig. 3). Table 2 shows the NPV, PPV, sensitivity, and accuracy of each model.

**Table 2:** Performance of Prediction Models for Predicting LNM of localized pancreatic ductal adenocarcinoma

Models	AUC (95% CI)	ACC	SEN	SPE	PPV	NPV
RS_male-muscle_	0.735 (0.645, 0.825)	0.624	0.559	0.845	0.786	0.653
RS_male-fat_	0.773 (0.689, 0.858)	0.684	0.525	0.931	0.886	0.659
RS_male-combined_	0.819 (0.743, 0.894)	0.726	0.712	0.810	0.792	0.734
RS_female-muscle_	0.811 (0.713, 0.908)	0.747	0.667	0.860	0.800	0.755
RS_female-fat_	0.895 (0.821, 0.970)	0.835	0.889	0.837	0.821	0.900
RS_female-combined_	0.924 (0.864, 0.984)	0.873	0.667	0.917	0.868	0.927

*LNM* lymph node metastasis, *RS* Radiomics signature, *AUC* area under the curve, *CI* confidence interval, *ACC* accuracy of leave one out cross-validation, *SEN* sensitivity, *SPE* specificity, *PPV* positive predictive value, *NPV* negative predictive value

## Discussion

Metabolic reprogramming is a sign of a malignant tumor, and tumor metastasis to lymph nodes requires abnormal metabolic transformation. The relationship between body composition that reflects host metabolism and lymph node metastasis of pancreatic cancer has not been reported. Hence, the purpose of this study is to design a gender-specific body composition radiomics method to predict LNM in patients with localized pancreatic cancer (LPDAC). Both adipose and muscle radiomics signature show good distinguishing ability for predicting LNM of LPDAC, and the female combined radiomics signature shows the best performance [AUC, 0.924].

According to the PDAC radiology reporting template proposed by the American Pancreatic Association and the Society of Abdominal Radiology, the diameter of regional LNs larger than 1 cm or other abnormal imaging signs are suspected to be involved [25]. However, this standard has performed poorly in many previous studies and our previous studies. All of these suggest that it is still very difficult for radiologists to predict lymph node metastasis.

At present, some studies have begun to use radiomics strategies to predict LNM in patients with pancreatic cancer. Li et al. reported a radiomics model with an AUC value of 0.91 in the validation set and 0.94 in the training set [9]. Liang et al. developed a radiomics nomogram with an AUC value of 0.80 in the primary cohort and 0.78 in the validation cohort [10]. Gao et al. established a radiomics model with good performance, which was similar to Liu et al. [11, 12]. Bian et al. directly focused on the lymph node itself, combined with artificial intelligence, and obtained the best performance (AUC, 0.92) [26]. However, these studies ignored the fact that pancreatic cancer is a systemic disease and the role of body composition changes in the prediction of LNM. Previous studies have shown that lymph nodes are considered a lipid-rich microenvironment, in which LNM tumor cells may give priority to the use of fatty acids as energy [15]. Rupert et al. reported that there is signal loop crosstalk between tumor-fat-muscle [27]. The deep-learning radiomics model established by An et al. showed that the model is most concerned with the tissue surrounding the tumor, rather than the tumor itself [28]. All of these suggest the value of body composition (adipose, muscle) in the assessment of LNM. Based on the above, this study focuses on body composition and uses a new quantitative radiomics strategy to explore the value of body composition-based radiomics in predicting the LNM of LPDAC.

This study was conducted on sexual dimorphism. As one of the earliest and most important advances in personalized medicine, sex-specific medicine has received great attention. It has been deeply studied in obesity, cancer, cardiovascular disease and other diseases [29]. There are significant differences in the distribution pattern and quality of adipose and muscle between sex [30–33]. This study found that Both male and female CT-measured body composition radiomics signatures have a certain predictive power on LNM of LPDAC. Among them, the female adipose signature showed the highest performance. Further analysis also showed that the predictive efficiency of body composition radiomics features in female patients was better than that in male patients. It is speculated that this may be related to the influence of sex-related hormones, and the underlying pathophysiological mechanism remains to be further investigated.

Although this study has tried to avoid the methodological issues of Radiomics in pancreatic tumors discussed by Bezzi C et al. [34], there are still several limitations in this study. First, the sample size of this study is small, especially for different gender groups. Second, a single slice of 2D features is used, rather than 3D features, based on the fact that the whole abdomen 3D segmentation of adipose and muscle or deep learning-based segmentation may be more representative of the body composition statue. Third, this study lacks an external validation of radiomics features. To obtain high-level evidence of clinical application, multicenter validation is required with larger sample size. Finally, clinical biochemical indicators such as Ca199 are not included as the purpose of this study is to focus on the predictive value of body components for LNM, which preliminarily proves the feasibility of body composition radiomics based on CT images to predict LNM of LPDAC. To further improve the accuracy of preoperative assessment of the lymph node status of pancreatic cancer, further work is expected to combine tumors, lymph nodes, and body components to develop a multidimensional predictive model.

In summary, this study demonstrated the feasibility of CT-measured body composition radiomics in predicting LNM of pancreatic cancer, with sexual dimorphism differences. It may provide a reference for personalized diagnosis and treatment of pancreatic cancer and related research on body composition in the future.

## Supplementary Information

**Additional file 1.**

## Data Availability

The datasets used and analysed during the current study are available from the corresponding author on reasonable request.

## Abbreviations

LNM: Lymph node metastasis
LPDAC: Localized pancreatic ductal adenocarcinoma
ROC: Receiver operating characteristic
AUC: Area under the curve
95%CI: 95% Confidence interval
