# Supplementary material for: CT-measured body composition radiomics predict lymph node metastasis in localized pancreatic ductal adenocarcinoma
Source: Discov Oncol. 2023 Feb 3;14:16. doi: 10.1007/s12672-023-00624-3 (PMC9898483; doi:10.1007/s12672-023-00624-3)
Supplement: Supplementary file 1 — Additional file 1. [file 12672_2023_624_MOESM1_ESM.pdf]

Radiomics features

May 18, 2017

1 First order statistics (19 features)

Notations:

$\mathbf{X}$ is an image of N voxels included in the ROI

P_i is the first order histogram with N_l discrete intensity levels, in which N_l is the number of non-zero bins

p_i is the normalized first order histogram and equal to $\frac{P_i}{\sum P_i}$ (This definition is the same for the following sections)

- **10Percentile**

The 10th percentile of $\mathbf{X}$.

- **90Percentile**

The 90th percentile of $\mathbf{X}$.

- **Energy**

$$energy = \sum_{i=1}^{N} (\mathbf{X}(i) + c)^2$$

Here, c is optional value, defined by "voxelArrayShift", which shifts the intensities to prevent negative values in $\mathbf{X}$. This ensures that voxels with the lowest gray values contribute the least to Energy, instead of voxels with gray level intensity closest to 0.

Energy is a measure of the magnitude of voxel values in an image. A larger values implies a greater sum of the squares of these values.

Note:

This feature is volume-confounded, a larger value of c increases the effect of volume-confounding.

- **Entropy**

$$entropy = -\sum_{i=1}^{N_l} p(i) \log_2 \big(p(i) + \epsilon\big)$$

Here, ϵ is an arbitrarily small positive number ($\approx 2.2 \times 10^{-16}$).

Entropy specifies the uncertainty/randomness in the image values. It measures the average amount of information required to encode the image values.

- **InterquartileRange**

$$interquartile\ range = \mathbf{P}_{75} - \mathbf{P}_{25}$$

Here $\mathbf{P}_{25}$ and $\mathbf{P}_{75}$ are the 25th and 75th percentile of the image array, respectively.

- **Kurtosis**

$$kurtosis = \frac{\mu_4}{\sigma^4} = \frac{\frac{1}{N}\sum_{i=1}^{N}(\mathbf{X}(i) - \bar{X})^4}{\left(\frac{1}{N}\sum_{i=1}^{N}(\mathbf{X}(i) - \bar{X})^2\right)^2}$$

Where μ_4 is the 4th central moment.

Kurtosis is a measure of the 'peakedness' of the distribution of values in the image ROI. A higher kurtosis implies that the mass of the distribution is concentrated towards the tail(s) rather than towards the mean. A lower kurtosis implies the reverse: that the mass of the distribution is concentrated towards a spike near the Mean value.

Related links:
https://en.wikipedia.org/wiki/Kurtosis

- **Maximum**

$$maximum = \max(\mathbf{X})$$

The maximum gray level intensity within the ROI.

- **Mean**

$$mean = \frac{1}{N}\sum_{i=1}^{N}\mathbf{X}(i)$$

The average gray level intensity within the ROI.

- **MeanAbsoluteDeviation**

$$MAD = \frac{1}{N}\sum_{i=1}^{N}|\mathbf{X}(i)-\bar{X}|$$

Mean Absolute Deviation is the mean distance of all intensity values from the Mean Value of the image array.

- **Median**

  The median gray level intensity within the ROI.

- **Minimum**

$$minimum = \min(\mathbf{X})$$

- **Range**

$$range = \max(\mathbf{X}) - \min(\mathbf{X})$$

The range of gray values in the ROI.

- **RobustMeanAbsoluteDeviation**

$$rMAD = \frac{1}{N_{10-90}}\sum_{i=1}^{N_{10-90}}|\mathbf{X}_{10-90}(i)-\bar{X}_{10-90}|$$

Robust Mean Absolute Deviation is the mean distance of all intensity values from the Mean Value calculated on the subset of image array with gray levels in between, or equal to the 10th and $90^{th} percentile$.

- **RootMeanSquared**

$$RMS = \sqrt{\frac{1}{N}\sum_{i=1}^{N}(\mathbf{X}(i)+c)^2}$$

Here, c is optional value, defined by "voxelArrayShift", which shifts the intensities to prevent negative values in $\mathbf{X}$. This ensures that voxels with the lowest gray values contribute the least to RMS, instead of voxels with gray level intensity closest to 0.

RMS is the square-root of the mean of all the squared intensity values. It is another measure of the magnitude of the image values. This feature is volume-confounded, a larger value of c increases the effect of volume-confounding.

- **Skewness**

$$skewness = \frac{\mu_3}{\sigma^3} = \frac{\frac{1}{N}\sum_{i=1}^{N}(\mathbf{X}(i)-\bar{X})^3}{\left(\sqrt{\frac{1}{N}\sum_{i=1}^{N}(\mathbf{X}(i)-\bar{X})^2}\right)^3}$$

Where μ_3 is the 3rd central moment.

Skewness measures the asymmetry of the distribution of values about the Mean value. Depending on where the tail is elongated and the mass of the distribution is concentrated, this value can be positive or negative.

Related links:

https://en.wikipedia.org/wiki/Skewness

- **StandardDeviation**

$$standard\ deviation = \sqrt{\frac{1}{N}\sum_{i=1}^{N}(\mathbf{X}(i)-\bar{X})^2}$$

Standard Deviation measures the amount of variation or dispersion from the Mean Value. By definition, $standard\ deviation = \sqrt{variance}$.

- **TotalEnergy**

$$total\ energy = V_{voxel}\sum_{i=1}^{N}(\mathbf{X}(i)+c)^2$$

Here, c is optional value, defined by "voxelArrayShift", which shifts the intensities to prevent negative values in $\mathbf{X}$. This ensures that voxels with the lowest gray values contribute the least to Energy, instead of voxels with gray level intensity closest to 0.

Total Energy is the value of Energy feature scaled by the volume of the voxel in cubic mm.

Note

This feature is volume-confounded, a larger value of c increases the effect of volume-confounding.

- **Uniformity**

$$uniformity = \sum_{i=1}^{N_l} p(i)^2$$

Uniformity is a measure of the sum of the squares of each intensity value. This is a measure of the heterogeneity of the image array, where a greater uniformity implies a greater heterogeneity or a greater range of discrete intensity values.

- **Variance**

$$variance = \frac{1}{N}\sum_{i=1}^{N}(\mathbf{X}(i) - \bar{X})^2$$

Variance is the the mean of the squared distances of each intensity value from the Mean value. This is a measure of the spread of the distribution about the mean. By definition, $variance = \sigma^2$.

2 Shape features 13 features

- **Compactness1**

$$compactness\ 1 = \frac{V}{\sqrt{\pi A^3}}$$

Similar to Sphericity, Compactness 1 is a measure of how compact the shape of the tumor is relative to a sphere (most compact). It is therefore correlated to Sphericity and redundant. It is provided here for completeness. The value range is $0 < compactness\ 1 \leq \frac{1}{6\pi}$, where a value of $\frac{1}{6\pi}$ indicates a perfect sphere.

By definition, $compactness\ 1 = \frac{1}{6\pi}\sqrt{compactness\ 2} = \frac{1}{6\pi}\sqrt{sphericity^3}$.

Note: This feature is correlated to Compactness 2, Sphericity and Spherical Disproportion. In the default parameter file provided in the “pyradiomics\bin“ folder, Compactness 1 and Compactness 2 are therefore disabled.

- **Compactness2**

$$compactness\ 2 = 36\pi\frac{V^2}{A^3}$$

Similar to Sphericity and Compactness 1, Compactness 2 is a measure of how compact the shape of the tumor is relative to a sphere (most compact). It is a dimensionless measure, independent of scale and orientation. The value range is $0 < compactness\ 2 \leq 1$, where a value of 1 indicates a perfect sphere.

By definition, $compactness\ 2 = (sphericity)^3$.

Note:

This feature is correlated to Compactness 1, Sphericity and Spherical Disproportion. In the default parameter file provided in the “pyradiomics\bin“ folder, Compactness 1 and Compactness 2 are therefore disabled.

- **Elongation**

Elongation is calculated using its implementation in SimpleITK, and is defined as:

$$elongation = \sqrt{\frac{\lambda_{\text{minor}}}{\lambda_{\text{major}}}}$$

Here, λ_{major} and λ_{minor} are the lengths of the largest and second largest principal component axes. The values range between 1 (where the cross section through the first and second largest principal moments is circle-like (non-elongated)) and 0 (where the object is a single point or 1 dimensional line).

- **Flatness**

Flatness is calculated using its implementation in SimpleITK, and is defined as:

$$\textit{flatness} = \sqrt{\frac{\lambda_{\text{least}}}{\lambda_{\text{major}}}}$$

Here, λ_{major} and λ_{least} are the lengths of the largest and smallest principal component axes. The values range between 1 (non-flat, sphere-like) and 0 (a flat object).

- **Maximum2DDiameterColumn**

Maximum 2D diameter (Column) is defined as the largest pairwise Euclidean distance between tumor surface voxels in the row-slice (usually the coronal) plane.

- **Maximum2DDiameterRow**

Maximum 2D diameter (Row) is defined as the largest pairwise Euclidean distance between tumor surface voxels in the column-slice (usually the sagittal) plane.

- **Maximum2DDiameterSlice**

Maximum 2D diameter (Slice) is defined as the largest pairwise Euclidean distance between tumor surface voxels in the row-column (generally the axial) plane.

- **Maximum3DDiameter**

Maximum 3D diameter is defined as the largest pairwise Euclidean distance between surface voxels in the ROI.

Also known as Feret Diameter.

- **SphericalDisproportion**

$$\textit{spherical disproportion} = \frac{A}{4\pi R^2} = \frac{A}{\sqrt[3]{36\pi V^2}}$$

Where R is the radius of a sphere with the same volume as the tumor, and equal to $\sqrt[3]{\frac{3V}{4\pi}}$.

Spherical Disproportion is the ratio of the surface area of the tumor region to the surface area of a sphere with the same volume as the tumor region, and by definition, the inverse of Sphericity. Therefore, the value range is $spherical\ disproportion \geq 1$, with a value of 1 indicating a perfect sphere.

Note: This feature is correlated to Compactness 1, Sphericity and Spherical Disproportion. In the default parameter file provided in the "pyradiomics\bin" folder, Compactness 1 and Compactness 2 are therefore disabled.

- **Sphericity**

$$sphericity = \frac{\sqrt[3]{36\pi V^2}}{A}$$

Sphericity is a measure of the roundness of the shape of the tumor region relative to a sphere. It is a dimensionless measure, independent of scale and orientation. The value range is $0 < sphericity \leq 1$, where a value of 1 indicates a perfect sphere (a sphere has the smallest possible surface area for a given volume, compared to other solids).

Note: This feature is correlated to Compactness 1, Compactness 2 and Spherical Disproportion. In the default parameter file provided in the "pyradiomics\bin" folder, Compactness 1 and Compactness 2 are therefore disabled.

- **SurfaceArea**

$$A = \sum_{i=1}^{N} \frac{1}{2}|\text{a}_i\text{b}_i \times \text{a}_i\text{c}_i|$$

Where:

N is the number of triangles forming the surface mesh of the volume (ROI)

a_ib_i and a_ic_i are the edges of the ith triangle formed by points a_i, b_i and c_i

Surface Area is an approximation of the surface of the ROI in mm2, calculated using a marching cubes algorithm.

References:

- Lorensen WE, Cline HE. Marching cubes: A high resolution 3D surface construction algorithm. ACM SIGGRAPH Comput Graph http://portal.acm.org/citation.cfm?doid=37402.37422 $1987; 21:163-9$.

- **SurfaceVolumeRatio**

$$surface\ to\ volume\ ratio = \frac{A}{V}$$

Here, a lower value indicates a more compact (sphere-like) shape. This feature is not dimensionless, and is therefore (partly) dependent on the volume of the ROI.

- **Volume**

The volume of the ROI is approximated by multiplying the number of voxels in the ROI by the volume of a single voxel.

3 GLCM features 28 features

Notations:

$\mathbf{P}(i, j)$ is the co-occurence matrix for δ (distance) and α (angle)

$p(i, j)$ is the normalized co-occurence matrix

N_g is the number of discrete intensity levels in the image

$p_x(i) = \sum_{j=1}^{N_g} P(i, j)$ is the marginal row probabilities

$p_y(j) = \sum_{i=1}^{N_g} P(i, j)$ is the marginal column probabilities

$\mu_x = \sum_{i=1}^{N_g} \sum_{j=1}^{N_g} P(i, j)i$ is the mean gray level intensity of p_x

$\mu_y = \sum_{i=1}^{N_g} \sum_{j=1}^{N_g} P(i, j)j$ is the mean gray level intensity of p_y

σ_x is the standard deviation of p_x

σ_y is the standard deviation of p_y

$p_{x+y}(k) = \sum_{i=1}^{N_g} \sum_{j=1}^{N_g} P(i, j)$, where $i + j = k$

$p_{x-y}(k) = \sum_{i=1}^{N_g} \sum_{j=1}^{N_g} P(i, j)$, where $\mid i - j \mid = k$

$HX = -\sum_{i=1}^{N_g} p_x(i) \log_2(p_x(i) + \epsilon)$ is the entropy of p_x

$HY = -\sum_{j=1}^{N_g} p_y(j) \log_2(p_y(j) + \epsilon)$ is the entropy of p_y

$HXY = -\sum_{i=1}^{N_g} \sum_{j=1}^{N_g} p(i, j) \log_2(p(i, j) + \epsilon)$ is the entropy of $p(i, j)$

$HXY1 = -\sum_{i=1}^{N_g} \sum_{j=1}^{N_g} p(i, j) \log_2(p_x(i)p_y(j) + \epsilon)$

$HXY2 = -\sum_{i=1}^{N_g} \sum_{j=1}^{N_g} p_x(i)p_y(j) \log_2(p_x(i)p_y(j) + \epsilon)$

- **Autocorrelation**

$$autocorrelation = \sum_{i=1}^{N_g} \sum_{j=1}^{N_g} p(i, j)ij$$

Autocorrelation is a measure of the magnitude of the fineness and coarseness of texture.

- **AverageIntensity**

$$\mu_x = \sum_{i=1}^{N_g} \sum_{j=1}^{N_g} p(i, j)i$$

Returns the mean gray level intensity of the i distribution.

Warning:

As this formula represents the average of the distribution of i, it is independent from the distribution of j. Therefore, only use this formula if the GLCM is symmetrical, where $p_x(i) = p_y(j)$, where $i = j$.

- **ClusterProminence**

$$cluster\ prominence = \sum_{i=1}^{N_g}\sum_{j=1}^{N_g} \big(i + j - \mu_x(i) - \mu_y(j)\big)^4 p(i,j)$$

Cluster Prominence is a measure of the skewness and asymmetry of the GLCM. A higher values implies more asymmetry about the mean while a lower value indicates a peak near the mean value and less variation about the mean.

- **ClusterShade**

$$cluster\ shade = \sum_{i=1}^{N_g}\sum_{j=1}^{N_g} \big(i + j - \mu_x(i) - \mu_y(j)\big)^3 p(i,j)$$

Cluster Shade is a measure of the skewness and uniformity of the GLCM. A higher cluster shade implies greater asymmetry about the mean.

- **ClusterTendency**

$$cluster\ tendency = \sum_{i=1}^{N_g}\sum_{j=1}^{N_g} \big(i + j - \mu_x(i) - \mu_y(j)\big)^2 p(i,j)$$

Cluster Tendency is a measure of groupings of voxels with similar gray-level values.

- **Contrast**

$$contrast = \sum_{i=1}^{N_g}\sum_{j=1}^{N_g} (i - j)^2 p(i,j)$$

Contrast is a measure of the local intensity variation, favoring values away from the diagonal ($i = j$). A larger value correlates with a greater disparity in intensity values among neighboring voxels.

- **Correlation**

$$correlation = \frac{\sum_{i=1}^{N_g}\sum_{j=1}^{N_g} p(i,j)ij - \mu_x(i)\mu_y(j)}{\sigma_x(i)\sigma_y(j)}$$

Correlation is a value between 0 (uncorrelated) and 1 (perfectly correlated) showing the linear dependency of gray level values to their respective voxels in the GLCM.

Note:

When there is only 1 discreet gray value in the ROI (flat region), σ_x and σ_y will be 0. In this case, the value of correlation will be a NaN.

- **DifferenceAverage**

$$\textit{difference average} = \sum_{k=0}^{N_g-1} k p_{x-y}(k)$$

Difference Average measures the relationship between occurrences of pairs with similar intensity values and occurrences of pairs with differing intensity values.

- **DifferenceEntropy**

$$\textit{difference entropy} = \sum_{k=0}^{N_g-1} p_{x-y}(k) \log_2 \big(p_{x-y}(k) + \epsilon\big)$$

Difference Entropy is a measure of the randomness/variability in neighborhood intensity value differences.

- **DifferenceVariance**

$$\textit{difference variance} = \sum_{k=0}^{N_g-1} (1 - DA)^2 p_{x-y}(k)$$

Difference Variance is a measure of heterogeneity that places higher weights on differing intensity level pairs that deviate more from the mean.

- **Dissimilarity**

$$\textit{dissimilarity} = \sum_{i=1}^{N_g} \sum_{j=1}^{N_g} |i - j| p(i,j)$$

Dissimilarity is a measure of local intensity variation defined as the mean absolute difference between the neighbouring pairs. A larger value correlates with a greater disparity in intensity values among neighboring voxels.

- **Energy**

$$\textit{energy} = \sum_{i=1}^{N_g} \sum_{j=1}^{N_g} \big(p(i,j)\big)^2$$

Energy (or Angular Second Moment)is a measure of homogeneous patterns in the image. A greater Energy implies that there are more instances of intensity value pairs in the image that neighbor each other at higher frequencies.

- **Entropy**

$$entropy = -\sum_{i=1}^{N_g}\sum_{j=1}^{N_g} p(i,j)\log_2\big(p(i,j)+\epsilon\big)$$

Entropy is a measure of the randomness/variability in neighborhood intensity values.

- **Homogeneity1**

$$homogeneity\ 1 = \sum_{i=1}^{N_g}\sum_{j=1}^{N_g}\frac{p(i,j)}{1+|i-j|}$$

Homogeneity 1 is a measure of the similarity in intensity values for neighboring voxels. It is a measure of local homogeneity that increases with less contrast in the window.

- **Homogeneity2**

$$homogeneity\ 2 = \sum_{i=1}^{N_g}\sum_{j=1}^{N_g}\frac{p(i,j)}{1+|i-j|^2}$$

Homogeneity 2 is a measure of the similarity in intensity values for neighboring voxels.

- **Id**

$$ID = \sum_{i=1}^{N_g}\sum_{j=1}^{N_g}\frac{p(i,j)}{1+|i-j|}$$

ID (inverse difference) is another measure of the local homogeneity of an image. With more uniform gray levels, the denominator will remain low, resulting in a higher overall value.

- **Idm**

$$IDM = \sum_{i=1}^{N_g}\sum_{j=1}^{N_g}\frac{p(i,j)}{1+|i-j|^2}$$

IDM (inverse difference moment) is a measure of the local homogeneity of an image. IDM weights are the inverse of the Contrast weights (decreasing exponentially from the diagonal $i = j$ in the GLCM).

- **Idmn**

$$IDMN = \sum_{i=1}^{N_g}\sum_{j=1}^{N_g}\frac{p(i,j)}{1+\left(\frac{|i-j|^2}{N_g^2}\right)}$$

IDMN (inverse difference moment normalized) is a measure of the local homogeneity of an image. IDMN weights are the inverse of the Contrast weights (decreasing exponentially from the diagonal $i = j$ in the GLCM). Unlike Homogeneity2, IDMN normalizes the square of the difference between neighboring intensity values by dividing over the square of the total number of discrete intensity values.

- **Idn**

$$IDN = \sum_{i=1}^{N_g}\sum_{j=1}^{N_g}\frac{p(i,j)}{1+\left(\frac{|i-j|}{N_g}\right)}$$

IDN (inverse difference normalized) is another measure of the local homogeneity of an image. Unlike Homogeneity1, IDN normalizes the difference between the neighboring intensity values by dividing over the total number of discrete intensity values.

- **Imc1**

$$IMC\ 1 = \frac{HXY - HXY1}{\max\{HX, HY\}}$$

- **Imc2**

$$IMC\ 2 = \sqrt{1 - e^{-2(HXY2 - HXY)}}$$

- **InverseVariance**

$$inverse\ variance = \sum_{i=1}^{N_g}\sum_{j=1}^{N_g}\frac{p(i,j)}{|i-j|^2}, i \neq j$$

- **MaximumProbability**

$$maximum\ probability = \max\big(p(i,j)\big)$$

Maximum Probability is occurrences of the most predominant pair of neighboring intensity values.

- **SumAverage**

$$sum\ average = \sum_{k=2}^{2N_g} p_{x+y}(k)k$$

Sum Average measures the relationship between occurrences of pairs with lower intensity values and occurrences of pairs with higher intensity values.

- **SumEntropy**

$$sum\ entropy = \sum_{k=2}^{2N_g} p_{x+y}(k) \log_2 \big(p_{x+y}(k) + \epsilon\big)$$

Sum Entropy is a sum of neighborhood intensity value differences.

- **SumSquares**

$$sum\ squares = \sum_{i=1}^{N_g} \sum_{j=1}^{N_g} (i - \mu_x)^2 p(i,j)$$

Sum of Squares or Variance is a measure in the distribution of neigboring intensity level pairs about the mean intensity level in the GLCM.

Warning:

This formula represents the variance of the distribution of i and is independent from the distribution of j. Therefore, only use this formula if the GLCM is symmetrical, where $p_x(i) = p_y(j)$, where $i = j$.

- **SumVariance**

$$sum\ variance = \sum_{k=2}^{2N_g} (k - SA)^2 p_{x+y}(k)$$

Sum Variance is a measure of heterogeneity that places higher weights on neighboring intensity level pairs that deviate more from the mean.

- **SumVariance2**

Using coefficients p_{x+y} and SumAvarage (SA) calculate and return the mean Sum Variance 2.

$$sum\ variance\ 2 = \sum_{k=2}^{2N_g} (k - SA)^2 p_{x+y}(k)$$

Sum Variance 2 is a measure of heterogeneity that places higher weights on neighboring intensity level pairs that deviate more from the mean.

This formula differs from SumVariance in that instead of subtracting the SumEntropy from the intensity, it subtracts the SumAvarage, which is the mean of intensities and not its entropy.

4 GLRLM features 16 features

Notations:

$\mathbf{P}(i, j \mid \theta)$ is the run length matrix of direction θ
$p(i, j \mid \theta)$ is the normalized run length matrix
N_g is the number of discrete intensity values in the image
N_r is the number of discrete run lengths in the image
N_p is the number of voxels in the image

- **GrayLevelNonUniformity**

$$GLN = \frac{\sum_{i=1}^{N_g} \left(\sum_{j=1}^{N_r} \mathbf{P}(i, j|\theta) \right)^2}{\sum_{i=1}^{N_g} \sum_{j=1}^{N_r} \mathbf{P}(i, j|\theta)}$$

GLN measures the similarity of gray-level intensity values in the image, where a lower GLN value correlates with a greater similarity in intensity values.

- **GrayLevelNonUniformityNormalized**

$$GLNN = \frac{\sum_{i=1}^{N_g} \left(\sum_{j=1}^{N_r} \mathbf{P}(i, j|\theta) \right)^2}{\sum_{i=1}^{N_g} \sum_{j=1}^{N_r} \mathbf{P}(i, j|\theta)^2}$$

GLNN measures the similarity of gray-level intensity values in the image, where a lower GLNN value correlates with a greater similarity in intensity values. This is the normalized version of the GLN formula.

- **GrayLevelVariance**

$$GLV = \sum_{i=1}^{N_g} \sum_{j=1}^{N_r} p(i, j|\theta)(i - \mu)^2$$

Here, $\mu = \displaystyle\sum_{i=1}^{N_g} \sum_{j=1}^{N_r} p(i, j|\theta) i$

GLV measures the variance in gray level intensity for the runs.

- **HighGrayLevelRunEmphasis**

$$HGLRE = \frac{\sum_{i=1}^{N_g} \sum_{j=1}^{N_r} \mathbf{P}(i, j|\theta) i^2}{\sum_{i=1}^{N_g} \sum_{j=1}^{N_r} \mathbf{P}(i, j|\theta)}$$

HGLRE measures the distribution of the higher gray-level values, with a higher value indicating a greater concentration of high gray-level values in the image.

- **LongRunEmphasis**

$$LRE = \frac{\sum_{i=1}^{N_g} \sum_{j=1}^{N_r} \mathbf{P}(i,j|\theta) j^2}{\sum_{i=1}^{N_g} \sum_{j=1}^{N_r} \mathbf{P}(i,j|\theta)}$$

LRE is a measure of the distribution of long run lengths, with a greater value indicative of longer run lengths and more coarse structural textures.

- **LongRunHighGrayLevelEmphasis**

$$LRHGLRE = \frac{\sum_{i=1}^{N_g} \sum_{j=1}^{N_r} \mathbf{P}(i,j|\theta) i^2 j^2}{\sum_{i=1}^{N_g} \sum_{j=1}^{N_r} \mathbf{P}(i,j|\theta)}$$

LRHGLRE measures the joint distribution of long run lengths with higher gray-level values.

- **LongRunLowGrayLevelEmphasis**

$$LRLGLRE = \frac{\sum_{i=1}^{N_g} \sum_{j=1}^{N_r} \frac{\mathbf{P}(i,j|\theta) j^2}{i^2}}{\sum_{i=1}^{N_g} \sum_{j=1}^{N_r} \mathbf{P}(i,j|\theta)}$$

LRLGLRE measures the joint distribution of long run lengths with lower gray-level values.

- **LowGrayLevelRunEmphasis**

$$LGLRE = \frac{\sum_{i=1}^{N_g} \sum_{j=1}^{N_r} \frac{\mathbf{P}(i,j|\theta)}{i^2}}{\sum_{i=1}^{N_g} \sum_{j=1}^{N_r} \mathbf{P}(i,j|\theta)}$$

LGLRE measures the distribution of low gray-level values, with a higher value indicating a greater concentration of low gray-level values in the image.

- **RunEntropy**

$$RE = -\sum_{i=1}^{N_g} \sum_{j=1}^{N_r} p(i,j|\theta) \log_2(p(i,j|\theta) + \epsilon)$$

Here, ϵ is an arbitrarily small positive number ($\approx 2.2 \times 10^{-16}$).

RE measures the uncertainty/randomness in the distribution of run lengths and gray levels. A higher value indicates more heterogeneity in the texture patterns.

- **RunLengthNonUniformity**

$$RLN = \frac{\sum_{j=1}^{N_r} \left(\sum_{i=1}^{N_g} \mathbf{P}(i,j|\theta) \right)^2}{\sum_{i=1}^{N_g} \sum_{j=1}^{N_r} \mathbf{P}(i,j|\theta)}$$

RLN measures the similarity of run lengths throughout the image, with a lower value indicating more homogeneity among run lengths in the image.

- **RunLengthNonUniformityNormalized**

$$RLNN = \frac{\sum_{j=1}^{N_r} \left(\sum_{i=1}^{N_g} \mathbf{P}(i,j|\theta) \right)^2}{\sum_{i=1}^{N_g} \sum_{j=1}^{N_r} \mathbf{P}(i,j|\theta)}$$

RLNN measures the similarity of run lengths throughout the image, with a lower value indicating more homogeneity among run lengths in the image. This is the normalized version of the RLN formula.

- **RunPercentage**

$$RP = \sum_{i=1}^{N_g} \sum_{j=1}^{N_r} \frac{\mathbf{P}(i,j|\theta)}{N_p}$$

RP measures the coarseness of the texture by taking the ratio of number of runs and number of voxels in the ROI.

Values are in range $\frac{1}{N_p} \leq RP \leq 1$, with higher values indicating a larger portion of the ROI consists of short runs (indicates a more fine texture).

- **RunVariance**

$$RV = \sum_{i=1}^{N_g} \sum_{j=1}^{N_r} p(i,j|\theta)(j-\mu)^2$$

Here, $\mu = \sum_{i=1}^{N_g} \sum_{j=1}^{N_r} p(i,j|\theta)j$

RV is a measure of the variance in runs for the run lengths.

- **ShortRunEmphasis**

$$SRE = \frac{\sum_{i=1}^{N_g} \sum_{j=1}^{N_r} \frac{\mathbf{P}(i,j|\theta)}{i^2}}{\sum_{i=1}^{N_g} \sum_{j=1}^{N_r} \mathbf{P}(i,j|\theta)}$$

SRE is a measure of the distribution of short run lengths, with a greater value indicative of shorter run lengths and more fine textural textures.

- **ShortRunHighGrayLevelEmphasis**

$$SRHGLE = \frac{\sum_{i=1}^{N_g}\sum_{j=1}^{N_r}\frac{\mathbf{P}(i,j|\theta)i^2}{j^2}}{\sum_{i=1}^{N_g}\sum_{j=1}^{N_r}\mathbf{P}(i,j|\theta)}$$

SRHGLE measures the joint distribution of shorter run lengths with higher gray-level values.

- **ShortRunLowGrayLevelEmphasis**

$$SRLGLE = \frac{\sum_{i=1}^{N_g}\sum_{j=1}^{N_r}\frac{\mathbf{P}(i,j|\theta)}{i^2j^2}}{\sum_{i=1}^{N_g}\sum_{j=1}^{N_r}\mathbf{P}(i,j|\theta)}$$

SRLGLE measures the joint distribution of shorter run lengths with lower gray-level values.

5 GLSZM features 16 features

Several notations:
$\mathbf{P}(i,j)$ is the size zone matrix
$p(i,j)$ is the normalized size zone matrix
N_g is the number of discrete intensity values in the image
N_s is the number of discrete zone sizes in the image
N_p is the number of voxels in the image

- **GrayLevelNonUniformity**

$$GLN = \frac{\sum_{i=1}^{N_g}\left(\sum_{j=1}^{N_s}\mathbf{P}(i,j)\right)^2}{\sum_{i=1}^{N_g}\sum_{j=1}^{N_s}\mathbf{P}(i,j)}$$

GLN measures the variability of gray-level intensity values in the image, with a lower value indicating more homogeneity in intensity values.

- **GrayLevelNonUniformityNormalized**

$$GLNN = \frac{\sum_{i=1}^{N_g}\left(\sum_{j=1}^{N_s}\mathbf{P}(i,j)\right)^2}{\sum_{i=1}^{N_g}\sum_{j=1}^{N_d}\mathbf{P}(i,j)^2}$$

GLNN measures the variability of gray-level intensity values in the image, with a lower value indicating a greater similarity in intensity values. This is the normalized version of the GLN formula.

- **GrayLevelVariance**

$$GLV = \sum_{i=1}^{N_g}\sum_{j=1}^{N_s} p(i,j)(i-\mu)^2$$

Here, $\mu = \sum_{i=1}^{N_g}\sum_{j=1}^{N_s} p(i,j)i$.

GLV measures the variance in gray level intensities for the zones.

- **HighGrayLevelZoneEmphasis**

$$HGLZE = \frac{\sum_{i=1}^{N_g}\sum_{j=1}^{N_s}\mathbf{P}(i,j)i^2}{\sum_{i=1}^{N_g}\sum_{j=1}^{N_s}\mathbf{P}(i,j)}$$

HGLZE measures the distribution of the higher gray-level values, with a higher value indicating a greater proportion of higher gray-level values and size zones in the image.

- **LargeAreaEmphasis**

$$LAE = \frac{\sum_{i=1}^{N_g}\sum_{j=1}^{N_s}\mathbf{P}(i,j)j^2}{\sum_{i=1}^{N_g}\sum_{j=1}^{N_s}\mathbf{P}(i,j)}$$

LAE is a measure of the distribution of large area size zones, with a greater value indicative of more larger size zones and more coarse textures.

- **LargeAreaHighGrayLevelEmphasis**

$$LAHGLE = \frac{\sum_{i=1}^{N_g}\sum_{j=1}^{N_s}\mathbf{P}(i,j)i^2j^2}{\sum_{i=1}^{N_g}\sum_{j=1}^{N_s}\mathbf{P}(i,j)}$$

LAHGLE measures the proportion in the image of the joint distribution of larger size zones with higher gray-level values.

- **LargeAreaLowGrayLevelEmphasis**

$$LALGLE = \frac{\sum_{i=1}^{N_g}\sum_{j=1}^{N_s}\frac{\mathbf{P}(i,j)j^2}{i^2}}{\sum_{i=1}^{N_g}\sum_{j=1}^{N_s}\mathbf{P}(i,j)}$$

LALGLE measures the proportion in the image of the joint distribution of larger size zones with lower gray-level values.

- **LowGrayLevelZoneEmphasis**

$$LGLZE = \frac{\sum_{i=1}^{N_g} \sum_{j=1}^{N_s} \frac{\mathbf{P}(i,j)}{i^2}}{\sum_{i=1}^{N_g} \sum_{j=1}^{N_s} \mathbf{P}(i,j)}$$

LGLZE measures the distribution of lower gray-level size zones, with a higher value indicating a greater proportion of lower gray-level values and size zones in the image.

- **SizeZoneNonUniformity**

$$SZN = \frac{\sum_{j=1}^{N_s} \left(\sum_{i=1}^{N_g} \mathbf{P}(i,j) \right)^2}{\sum_{i=1}^{N_g} \sum_{j=1}^{N_s} \mathbf{P}(i,j)}$$

SZN measures the variability of size zone volumes in the image, with a lower value indicating more homogeneity in size zone volumes.

- **SizeZoneNonUniformityNormalized**

$$SZNN = \frac{\sum_{j=1}^{N_s} \left(\sum_{i=1}^{N_g} \mathbf{P}(i,j) \right)^2}{\sum_{i=1}^{N_g} \sum_{j=1}^{N_d} \mathbf{P}(i,j)^2}$$

SZNN measures the variability of size zone volumes throughout the image, with a lower value indicating more homogeneity among zone size volumes in the image. This is the normalized version of the SZN formula.

- **SmallAreaEmphasis**

$$SAE = \frac{\sum_{i=1}^{N_g} \sum_{j=1}^{N_s} \frac{\mathbf{P}(i,j)}{j^2}}{\sum_{i=1}^{N_g} \sum_{j=1}^{N_s} \mathbf{P}(i,j)}$$

SAE is a measure of the distribution of small size zones, with a greater value indicative of more smaller size zones and more fine textures.

- **SmallAreaHighGrayLevelEmphasis**

$$SAHGLE = \frac{\sum_{i=1}^{N_g} \sum_{j=1}^{N_s} \frac{\mathbf{P}(i,j)i^2}{j^2}}{\sum_{i=1}^{N_g} \sum_{j=1}^{N_s} \mathbf{P}(i,j)}$$

SAHGLE measures the proportion in the image of the joint distribution of smaller size zones with higher gray-level values.

- **SmallAreaLowGrayLevelEmphasis**

$$SALGLE = \frac{\sum_{i=1}^{N_g} \sum_{j=1}^{N_s} \frac{\mathbf{P}(i,j)}{i^2 j^2}}{\sum_{i=1}^{N_g} \sum_{j=1}^{N_s} \mathbf{P}(i,j)}$$

SALGLE measures the proportion in the image of the joint distribution of smaller size zones with lower gray-level values.

- **ZoneEntropy**

$$ZE = -\sum_{i=1}^{N_g} \sum_{j=1}^{N_s} p(i,j) \log_2(p(i,j) + \epsilon)$$

Here, ϵ is an arbitrarily small positive number ($\approx 2.2 \times 10^{-16}$).

ZE measures the uncertainty/randomness in the distribution of zone sizes and gray levels. A higher value indicates more heterogeneneity in the texture patterns.

- **ZonePercentage**

$$ZP = \sum_{i=1}^{N_g} \sum_{j=1}^{N_s} \frac{\mathbf{P}(i,j)}{N_p}$$

ZP measures the coarseness of the texture by taking the ratio of number of zones and number of voxels in the ROI.

Values are in range $\frac{1}{N_p} \leq ZP \leq 1$, with higher values indicating a larger portion of the ROI consists of small zones (indicates a more fine texture).

- **ZoneVariance**

$$ZV = \sum_{i=1}^{N_g} \sum_{j=1}^{N_s} p(i,j)(j - \mu)^2$$

Here, $\mu = \sum_{i=1}^{N_g} \sum_{j=1}^{N_s} p(i,j)j$

ZV measures the variance in zone size volumes for the zones.
